# Characterization of the complete chloroplast genome of *Polyspora tiantangensis* (Theaceae), an endemic and endangered species in southwestern China

**DOI:** 10.1080/23802359.2021.1884013

**Published:** 2021-03-11

**Authors:** Zhi-Feng Fan, Shao-Juan Qian, Yong-Hong Zhang, Chang-Le Ma

**Affiliations:** a School of Landscape Architecture and Horticulture Sciences, Southwest Forestry University, Kunming, China; b School of Life Sciences, Yunnan Normal University, Kunming, China; c Southwest Landscape Engineering Technology Research Center of State Forestry and Grassland Administration, Kunming, China

**Keywords:** *Polyspora tiantangensis*, chloroplast genome, endangered species, phylogenetic analysis

## Abstract

*Polyspora tiantangensis* (Theaceae) is an endangered woody plant in southwestern China. In this study, we assembled complete chloroplast (cp) genome of *P. tiantangensis* based on the Illumina reads. The cp genome of *P. tiantangensis* is 157,057 bp in length, including a large single-copy (LSC) region of 86,593 bp and a small single-copy (SSC) region of 18,284 bp, separated by two inverted repeat (IR) regions of 26,090 bp each. The cp genome encoded 132 genes including 87 protein-coding genes, 37 tRNA genes, and eight rRNA genes. The GC content of cp genome of *P. tiantangensis* is 37.3%. A total of 68 SSRs were discovered. Phylogenetic analysis of cp genomes from 26 species of Theaceae revealed that all species of *Polyspora* formed one monophyletic clade and *P. tiantangensis* was closely related with its congeneric species *P. longicarpa* with 100% bootstrap value.

*Polyspora tiantangensis* (L. L. Deng and G. S. Fan) S. X. Yang (Theaceae), an endangered evergreen woody plant, first reported its discovery in Tiantangshan state-owned forest farm of Yunnan province in 1999, published as a new species, named as *Gordonia tiantangensis* (Deng and Fan 1999). Since the taxonomy retreatment of the genus *Gordonia* by morphology and molecular evidences (Yang et al. 2004; Yang 2005; Bartholomew and Tienlu 2007), *Gordonia tiantangensis* was transferred to genus *Polyspora* with other species distribute in East Asia, and revised its name as *P. tiantangensis*. The species is narrowly distributed in broad-leaved evergreen forests in western Yunnan Province (Ma et al. 2015). It has beautiful tree shape, large, and white flowers blooming from October to January. With strong ecological adaptability to mountainous region, *P. tiantangensis* and other species of *Polyspora* native to China have been recommended as ornamental plants for urban and rural greening works in southern China (Ma et al. 2015). Here, the complete chloroplast (cp) genome of *P. tiantangensis* was characterized to provide genomic resource for further conservation genetics and phylogenetic analysis.

The fresh leaves of *P. tiantangensis* were collected from Tiantang Mountain, Lancangjiang Nature Reserve, Changning County of Yunnan Province (24°56′57″N, 99°37′46″E). The voucher specimen (Ma 15701) was deposited at Herbarium of Southwest Forestry University (SWFU). A sequence library for *P. tiantangensis* was generated using the Illumina HiSeq 2500-PE150 platform (Illumina, San Diego, CA). High quality clean reads were obtained using NGS QC Toolkit_v2.3.3 with default parameters (Patel and Jain 2012). The plastome was de novo assembled by NOVOPlasty (Dierckxsens et al. 2017). The cp genome was annotated with the online annotation tool GeSeq (Tillich et al. 2017).

The total cp genome size of *P. tiantangensis* is 157,057 bp (GenBank accession no. MT816506), containing a large single-copy (LSC) region of 86,593 bp and a small single-copy (SSC) region of 18,284 bp, separated by two inverted repeat (IR) regions of 26,090 bp. It contained 132 genes, including 87 protein-coding genes, eight rRNA genes, and 37 tRNA genes. The base compositions of the cp genome were uneven (31.1% A, 19.0% C, 18.3% G, and 31.6% T), with an overall GC content of 37.3%. IMEx (Mudunuri and Nagarajaram 2007) was used to identify the SSRs with minimum repeat number set to 10, 5, 4, 3, 3, and 3. A total of 68 SSRs were discovered, the numbers of mono-, di-, tri-, tetra-, penta-, and hexa-nucleotides SSRs are 54, 4, 0, 10, 0, and 0, respectively.

To clarify the phylogenetic position of *P. tiantangensis*, 26 published cp genomes from Theaceae were aligned by using MAFFT 7.308 (Katoh and Standley 2013) with *Stewartia sinensis* (MH737738) and *Hartia laotica* (NC041509) as outgroups. The maximum-likelihood (ML) tree was constructed with RAxML version 8 (Stamatakis 2014) and the clade support was estimated using 1000 bootstrap replicates. The phylogenetic tree showed that six species of *Polyspora* formed one monophyletic clade with 100% bootstrap value (Figure 1) and *P. tiantangensis* was closely related with its congeneric species, *P. longicarpa*. The complete cp genome of *P. tiantangensis* will provide useful resource for further study on the phylogeny, conservation genetics and molecular breeding.

**Figure 1.**
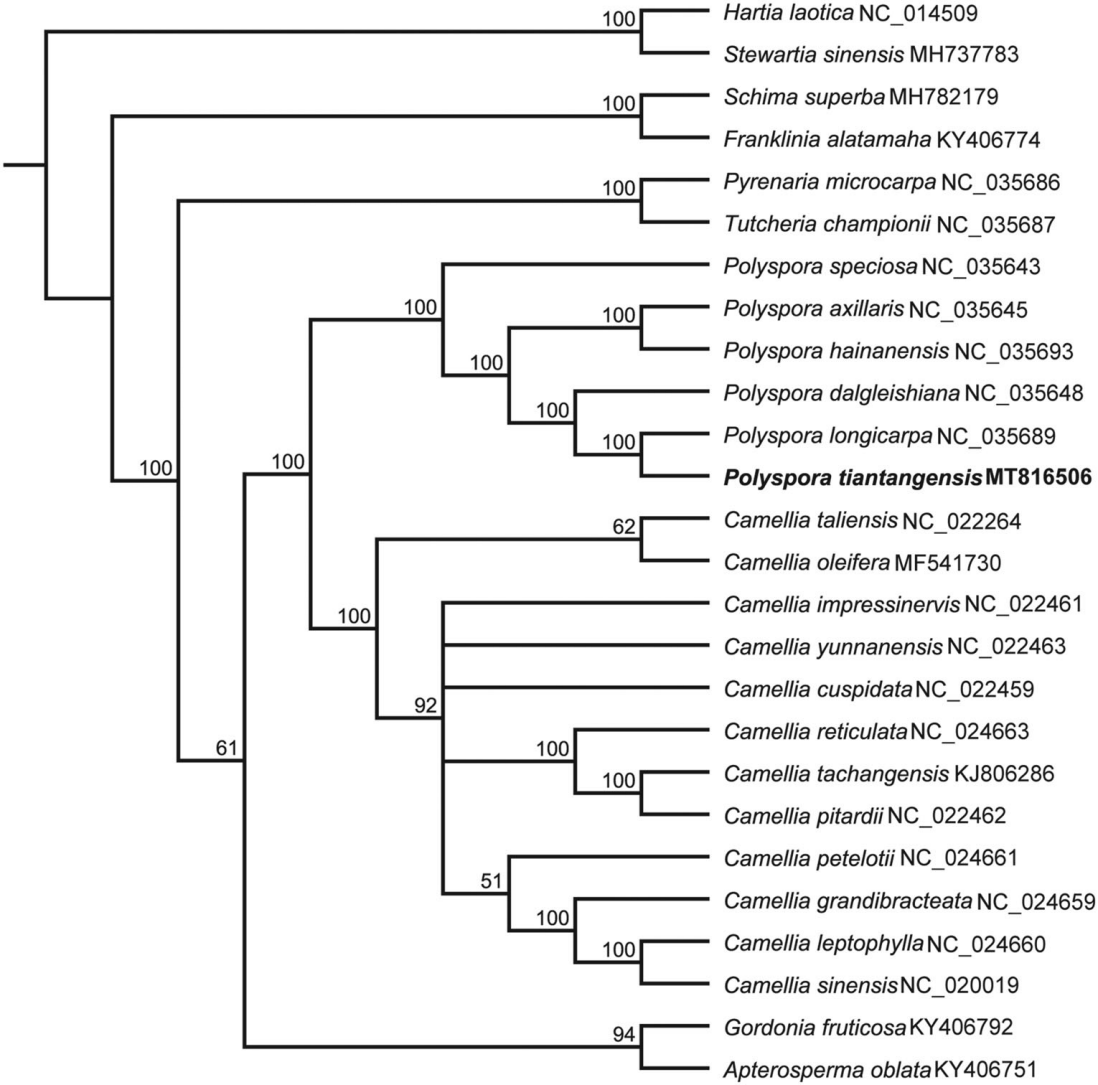
Molecular phylogenetic tree of 26 species of Theaceae based on complete plastome sequences with maximum-likelihood analysis.

## Data Availability

The data that support the findings of this study are openly available in NCBI at https://www.ncbi.nlm.nih.gov/, reference number: [MT816506] and [SRR13108242], or available from the corresponding author.
